# Discordantly Low HbA1c in Patients Receiving Dapsone: Prevalence and Associations with Hemolysis and Methemoglobinemia

**DOI:** 10.5812/ijem-166723

**Published:** 2026-04-27

**Authors:** Teena Chandran, Mohammed Anwar Hussain, Bhavesh Patel, Riddhi Dasgupta, Anu Anna George, Leni George, Dincy Peter, Felix K Jebasingh, Nihal Thomas

**Affiliations:** 1 Department of Endocrinology, Diabetes and Metabolism, Christian Medical College, Vellore, India; 2 Department of Dermatology, Christian Medical College, Vellore, India; 3 University of Melbourne, Melbourne, Australia; 4 Centre for Stem Cell Research, Christian Medical College, Vellore, India

**Keywords:** Dapsone, Diabetes Mellitus, Hemolysis, Methemoglobinemia, Glycosylated Hemoglobin

## Abstract

**Background:**

Glycosylated hemoglobin (HbA1c) is widely used for the diagnosis and long-term monitoring of diabetes mellitus (DM). However, its accuracy may be compromised by conditions and medications that alter erythrocyte lifespan or hemoglobin structure. Dapsone, commonly used in the treatment of Hansen’s disease, has been reported to cause spuriously low HbA1c values, but systematic data on its prevalence and underlying mechanisms remain limited.

**Objectives:**

The objectives of this study were to (1) determine the prevalence of discordantly low HbA1c levels in patients receiving dapsone therapy, including individuals with and without DM; (2) explore associations between discordant HbA1c and markers of hemolysis and methemoglobinemia; and (3) compare measured HbA1c values obtained by high-performance liquid chromatography (HPLC) with expected HbA1c values derived from fructosamine using the glycosyl gap.

**Methods:**

This retrospective cross-sectional study included 30 patients receiving oral dapsone for Hansen’s disease and 10 healthy controls. HbA1c was measured using ion-exchange HPLC and compared with expected HbA1c values calculated from serum fructosamine. Parameters related to hemolysis and methemoglobinemia were assessed. Discordant HbA1c was defined by a negative glycosyl gap. Subgroup analyses were exploratory.

**Results:**

Among patients receiving dapsone, 16 had DM and 14 were normoglycemic. Discordantly low HbA1c was observed in 27 of 30 patients (90%), including 15 of 16 patients with DM and 12 of 14 without diabetes. Markers of hemolysis and methemoglobinemia showed trends consistent with biological plausibility but did not demonstrate statistically significant differences sufficient to establish causality.

**Conclusions:**

A high prevalence of discordantly low HbA1c was observed among patients receiving dapsone therapy, highlighting an important clinical pitfall in the interpretation of HbA1c. While hemolysis and methemoglobinemia remain biologically plausible contributors, these findings should be considered hypothesis-generating. Clinicians should exercise caution when interpreting HbA1c in patients treated with dapsone and consider alternative glycemic markers such as fructosamine.

## 1. Background

Dapsone (4, 4`-diaminodiphenylsulfone) is a common medication used in the treatment of Hansen’s disease. It is used for prophylaxis against Pneumocystis carinii and toxoplasma infections and as an adjuvant in antimalarial therapy. It is also used to treat dermatological conditions such as pemphigus vulgaris, IgA pemphigus, bullous pemphigoid, bullous lesions in SLE, Dermatitis herpetiformis, Sweet syndrome, and pyoderma gangrenosum (1). Diabetes mellitus (DM) is a major cause of mortality and morbidity in the Indian subcontinent (2). Long-term complications of DM include both microvascular and macrovascular complications. Therefore, optimal glycemic control is essential to prevent or delay complications related to chronic hyperglycemia (3). Since 1980, glycated hemoglobin (HbA1c) has been used clinically as a test to assess glycemic status. It is a useful marker of chronic hyperglycemia that reflects blood glucose levels over a two- to three-month duration (4-6). Other chronic glycemic markers include fructosamine assay and 1,5-anhydroglucitol (7, 8). These tests play a critical role in the management of DM, as the levels of these markers in the blood are directly proportional to microvascular complications and, to a lesser extent, macrovascular complications. Hemoglobin (Hb) is a tetramer formed of two alpha and two beta globin chains. With chronic exposure to high glucose levels, Hb is non-enzymatically glycated at different sites, wherein glucose is adsorbed onto the N-terminal valine residue of the beta chain of Hb. There is sufficient evidence to suggest that HbA1c levels reflect the exposure to glucose levels to which red blood cells are exposed during their lifespan. HbA1c can be estimated using two types of methodologies: separation of hemoglobin methods, which include high-performance liquid chromatography (HPLC), capillary electrophoresis, and affinity chromatography techniques, and chemical methods, such as immunoassay and enzymatic assay techniques (9).

According to the ADA 2025, a diagnostic HbA1c test should be performed using a method approved by the National Glycohemoglobin Standardization Program (NGSP) (10). Moreover, the assay should be standardized using the Diabetes Control and Complications Trial (DCCT) reference assay. The HbA1c cutoff for the diagnosis of DM is > 6.5% (11). Currently, HbA1c assays are standardized globally. Though the assay has been standardized, there are many factors, including medications, that may interfere with the HbA1c assay, thereby altering its value. Uremia, hypertriglyceridemia, hyperbilirubinemia, chronic alcohol ingestion, salicylate ingestion, citric acid ingestion, and opiate addiction all interfere with certain assays, causing spuriously high HbA1c levels. Medications such as hydroxyurea and dapsone have been shown to cause low HbA1c values (12). Hence, one should interpret HbA1c with caution under these circumstances (13). Dapsone causes oxidative promotion of hemoglobin to methemoglobin, which may interfere with the HPLC assay, thereby causing low HbA1c levels. Second, it is postulated that dapsone reduces the lifespan of red cells, independent of its hemolytic effect. The potential lowering effect of HbA1c by dapsone is clinically significant in patients with associated diabetes (14). Dapsone has been reported to reduce HbA1c levels to a larger degree in various case reports (15). However, there are limited series to support the claim of falsely lowered HbA1c levels in patients receiving dapsone (16, 17). Dapsone is metabolized via the hepatic route through the cytochrome P450 pathway, which can cause hemolysis and methemoglobinemia. Dapsone-induced hemolysis is common in patients with G6PD (Glucose 6 phosphate dehydrogenase) deficiency due to the depletion of glutathione reserves in red blood cells (18). Hemolysis causes a reduction in erythrocyte lifespan and, hence, a falsely lower HbA1c (19). There is also a paucity of evidence regarding dapsone-associated methemoglobinemia. The spectrum of signs and symptoms varies from central or peripheral cyanosis (methemoglobin levels of approximately 15%) to headache and fatigue (methemoglobin levels of 30 - 45%) to cardiac arrhythmias (at 60%) and death (above 70%) (20, 21).

Despite these reports, the existing evidence is largely limited to isolated case reports and small case series. Systematic data on the prevalence of discordantly low HbA1c in patients receiving dapsone — particularly in cohorts including both diabetic and non-diabetic individuals — remain limited. In addition, the relative contribution of proposed mechanisms, such as haemolysis and methemoglobinemia, has not been adequately evaluated using objective biochemical markers. There are also limited comparative data between measured HbA1c and alternative glycaemic markers such as fructosamine in this clinical context.

## 2. Objectives

The objectives of this study were:

- To determine the prevalence of discordantly low HbA1c levels in patients receiving dapsone therapy, including both individuals with DM and normoglycemia.

- To explore potential mechanisms underlying discordant HbA1c values, with a specific focus on markers of hemolysis and methemoglobinemia.

- To compare measured HbA1c values obtained by HPLC with expected HbA1c values derived from fructosamine, and to evaluate the glycosyl gap as an alternative marker of glycemic status in patients receiving dapsone.

## 3. Methods

This was a retrospective cross-sectional study conducted in the Departments of Endocrinology, Diabetes and Metabolism, and Dermatology, Christian Medical College, Vellore, India, after approval by the Institutional Review Board (IRB No: 10515, dated 01.02.2017). Patients receiving dapsone therapy for Hansen’s disease were identified through a retrospective review of institutional medical records from 2018 to 2019. All patients received oral dapsone as part of standard multidrug therapy for Hansen’s disease. All patients were screened for G6PD deficiency prior to initiation of dapsone therapy. The prescribed dose was 100 mg daily in accordance with institutional and national treatment guidelines. For patients with multi-bacillary leprosy, dapsone was administered at 100 mg orally daily for 12 months, whereas for those with paucibacillary leprosy, dapsone was administered at 100 mg orally daily for 6 months. Eligible patients were included consecutively based on the availability of complete clinical and biochemical data. Following an informed consent, a total of 30 patients on dapsone therapy and 10 healthy controls of Asian Indian origin were included in the study. Healthy controls were individuals without known DM, not receiving dapsone therapy, and were included to establish baseline glycosyl gap parameters in the absence of dapsone exposure. These patients were retrospectively chosen from the database of the Department of Endocrinology, Diabetes and Metabolism.

### 3.1. Biochemical Measurements

The biochemical parameters were measured using the following assays. Fasting and postprandial plasma glucose levels were measured by the UV enzymatic hexokinase and G6PDH methods. HbA1c was estimated using the HPLC, ion-exchange method (Bio-Rad Variant II Turbo). Serum fructosamine was measured by a colorimetric method (Beckman AU 5800, Cobas 8000). In addition, parameters of hemolysis, including lactate dehydrogenase (LDH), reticulocyte count, and mean corpuscular volume (MCV), as well as methemoglobin, were estimated. Lactate dehydrogenase was measured using the International Federation of Clinical Chemistry (IFCC) method. Reticulocyte count and MCV were measured using automated hematology analyzers, and methemoglobin was measured by co-oximetry

### 3.2. Assessment of Discordant HbA1c

The measured HbA1c values were compared with expected HbA1c values derived from serum fructosamine levels. The expected HbA1c (%) was calculated using the following formula: Expected HbA1c (%) = 0.017 × fructosamine (mmol/L) + 1.61. The glycosyl gap (GG) was calculated as: Glycosyl gap (GG) = measured HbA1c − expected HbA1c (22). HbA1c levels were considered discordant when fructosamine and expected HbA1c values were higher than the measured HbA1c, and the glycosyl gap was negative. HbA1c levels were considered concordant when fructosamine and expected HbA1c values were similar to the measured HbA1c, and the glycosyl gap was positive.

### 3.3. Inclusion and Exclusion Criteria

Patients receiving dapsone therapy for Hansen’s disease were identified through retrospective review of institutional medical records. Patients with known hemoglobinopathies, chronic liver disease, chronic kidney disease, or those receiving medications known to interfere with HbA1c estimation (including sulfasalazine, ribavirin, and antiretroviral drugs) were excluded from the study.

### 3.4. Sample Size Justification

Based on prior institutional observations suggesting that approximately 70% of patients receiving dapsone therapy have discordantly low HbA1c values, the expected prevalence (p) was assumed to be 70%. This estimate was informed by a previously published single-center retrospective cohort study from our institution that demonstrated a prevalence of approximately 70% for discordantly low HbA1c among patients with DM receiving dapsone therapy (13). Using a confidence level of 95% and a precision (d) of ±12%, the sample size was estimated using the formula n = 4pq/d², where q = 1 − p. This yielded an estimated sample size of approximately 30 patients, which formed the study cohort.

A formal power analysis was not performed, as the study was designed primarily to estimate the prevalence of discordantly low HbA1c among patients receiving dapsone therapy rather than to detect statistically significant differences in subgroup or mechanistic analyses. Accordingly, the sample size was calculated to estimate prevalence and was not intended to provide adequate power for robust subgroup or mechanistic analyses.

### 3.5. Statistical Analysis

Statistical analysis was performed using SPSS version 23.0 software. Continuous variables were expressed as mean ± standard deviation (SD), and categorical variables were expressed as percentages. The normality of continuous variables was assessed prior to analysis. Independent sample *t*-tests were used to compare the means of continuous variables, and Fisher’s exact test was used to compare categorical variables. Statistical significance was set at P < 0.05. Subgroup analyses, particularly comparisons involving small groups such as the concordant HbA1c group, were performed in an exploratory manner and are presented descriptively. These analyses were intended to identify trends rather than to draw definitive statistical conclusions.

## 4. Results

A total of 30 patients who were on dapsone therapy and 10 healthy controls were included in the study. All patients on dapsone were treated for Hansen’s disease, and none had G6PD deficiency. Of the 30 patients, 16 had DM, and 14 did not. Compared to healthy controls, those on dapsone therapy had discordant HbA1c levels (glycosyl gap- -1.3 +1 vs. 0.2+0.1, p- 0.01). This suggests that a significant number of patients taking dapsone had falsely low HbA1c levels (Table 1). 

**Table 1.** Comparison of Patients on Dapsone and Normal Healthy Controls ^a^

Variables	Patients on Dapsone; (N = 30)	Normal Healthy Controls; (N = 10)	P-Value ^b^
**Age (y)**	45.4 ± 5	28 ± 8	0.01
**BMI (kg/m** ^**2**^ **)**	24.2 ± 4.2	19.6 ± 2.5	0.01
**Measured HbA1c (%)**	4.7 ± 1	5.1 ± 0.2	0.05
**Fructosamine (mmol/L)**	264 ± 58	200 ± 13	0.01
**Expected HBA1C (%)**	6 ± 1	5 ± 0.2	0.01
**Glycosyl Gap (%)**	-1.3 ± 1	0.2 ± 0.1	0.01

^a^ Values are as expressed as mean ± SD.

^b^ Significance was set at P < 0.05.

Of the 30 patients on dapsone, 27 had discordant HbA1c (DH) values compared to the corresponding fructosamine levels and glycosyl gap, and 3 had concordant HbA1c (CH) (Table 2). Among those with diabetes (N-16), 15 (94%) had DH, and among those without diabetes (N-14), 12 (86%) had DH. When patients with CH and DH were compared, those with CH were 5 years younger than those with DH. Patients with DH had a higher BMI than those with CH (P = 0.03). There was a statistically significant difference between CH and DH for the measured and estimated HbA1c levels, as well as the glycosyl gap. The MCV, reticulocyte counts, and LDH levels in DH were higher than those in CH, although the difference was not statistically significant, suggesting that hemolysis is a possible indicator of low HbA1c levels in patients taking dapsone (Table 3). The MCV levels showed a trend toward statistical significance (P = 0.09), although other markers of hemolysis were not significant. In addition, DH had marginally higher methemoglobin levels than CH, which was not statistically significant (P = 0.2).

**Table 2.** Discordant and Concordant HbA1c Levels in Comparison with Fructosamine Levels and the Glycosyl Gap ^a^

HbA1c	Patients with DM; (N = 16)	Patients with Normoglycemia; (N = 14)
**Discordance**	15 (94)	12 (86)
**Concordance**	1 (6)	2 (14)
**Total**	16 (100)	14 (100)

Abbreviation: DM, diabetes mellitus.

^a^ Values are as expressed as No. (%).

**Table 3.** Comparison Between the CH vs. DH [Concordant HbA1c, Discordant HbA1c] ^a^

Variables	CH (N = 3)	DH (N = 27)	P-Value
**Age (y)**	50 ± 8	44.9 ± 12.3	0.4
**BMI (kg/m** ^**2**^ **)**	21.6 ± 1.3	24.6 ± 4.3	0.03 ^b^
**Measured HbA1c (%)**	5.9 ± 0.4	4.6 ± 1.0	0.01 ^b^
**Fructosamine (mmol/L)**	242.6 ± 14.2	268.9 ± 62.9	0.01 ^b^
**Expected HBA1C (%)**	5.5 ± 0.11	6.1 ± 1.0	0.01 ^b^
**Glycosyl Gap (%)**	0.3 ± 0.3	-1.5 ± 0.8	0.01 ^b^
**Hemoglobin(g/mL)**	11.7 ± 3.1	11.5 ± 1.8	0.9
**MCV(Fl)**	84.2 ± 4.0	90.4 ± 7.2	0.09
**Reticulocyte (%)**	3.3 ± 3.4	3.2 ± 1.7	0.9
**LDH (U/L)**	549.7 ± 400.6	593.3 ± 257.7	0.9
**Methemoglobin(g/mL)**	2 ± 0.0	2.6 ± 2.3	0.2

^a^ Values are as expressed as mean ± SD.

^b^ Significance was set at P < 0.05.

All baseline parameters, except for expected HbA1c and fructosamine levels, were comparable between patients with and without diabetes. The mean fructosamine levels were 283.7 ± 72.3 (mmol/L) and 242.6 ± 24.2 (mmol/L), respectively. (P = 0.03) (Table 3). Among those without DM, DH had much lower HbA1c levels than CH, which was statistically significant (P = 0.01). Patients with DH were much younger and had a higher BMI than those with CH. There was no significant difference between CH and DH in the hemolysis parameters in patients without DM. In addition, the methemoglobin levels were similar in the CH and DH groups (Table 4 and Table 5). 

**Table 4.** Comparison Between Patients with and Without Diabetes Mellitus ^a^

Variables	Diabetes Group; N = 16	Without Diabetes Group; N = 14	P-Value
**Age (y)**	43.6 ± 12.2	46.9 ± 11.9	0.5
**BMI (kg/m** ^**2**^ **)**	25.0 ± 5	23.4 ± 2.9	0.3
**Measured HbA1c (%)**	5.0 ± 1.2	4.4 ± 0.7	0.1
**Fructosamine (mmol/L)**	283.7 ± 72.3	242.6 ± 24.2	0.04 ^b^
**Expected HbA1c (%)**	6.4 ± 1.2	5.6 ± 0.4	0.04 ^b^
**Glycosyl Gap (%)**	-1.4 ± 1	-1.2 ± 0.8	0.6
**Hemoglobin (g/dL)**	11.6 ± 1.08	11.5 ± 2.5	1
**MCV (fL)**	88.5 ± 7	90.8 ± 7.2	0.9
**Reticulocyte (%)**	2.9 ± 1.4	3.8 ± 2.4	0.7
**LDH (U/L)**	608.4 ± 262.9	571.9 ± 276.2	0.2
**Methemoglobin (g/mL)**	2 ± 0.2	3 ± 2.9	0.2

^a^ Values are as expressed as mean ± SD.

^b^ Significance was set at P < 0.05.

**Table 5.** Comparison Between CH and DH Among Those Without Diabetes Mellitus

Variables	CH (N = 2)	DH (N = 12)	P-Value
**Age (y)**	53 ± 9.8	42 ± 12.3	0.3
**BMI (Kg/m** ^**2**^ **)**	20.9 ± 1.2	23.8 ± 2.9	0.8
**HbA1c (%)**	5.6 ± 0.1	4.2 ± 0.5	0.01 ^b^
**Fructosamine (mmol/L)**	231.5 ± 12	244.4 ± 25.5	0.3
**Expected HBA1C (%)**	5.5 ± 0.1	5.7 ± 0.5	0.2
**Glycosyl Gap (%)**	0.1 ± 0.2	-1.4 ± 0.6	0.01 ^b^
**Hemoglobin(g/mL)**	11.2 ± 4.3	11.6 ± 2.5	0.9
**MCV (fL)**	86.5 ± 0.5	88.9 ± 7.7	0.3
**Reticulocyte (%)**	3.9 ± 4.5	3.3 ± 2.3	0.9
**LDH**	694 ± 442.6	594 ± 249.6	0.8
**Methemoglobin(g/mL)**	2 ± 0.0	2 ± 0.2	0.3

^a^ Values are as expressed as mean ± SD.

^b^ Significance was set at P < 0.05.

In addition, in patients with DM, the hemolytic parameters and methemoglobin levels were higher in the DH group than in the CH group. Comparisons between the groups were not performed because there was only one patient in the CH group. Of those with DM, 15 were already known to have DM and were treated for the same. One patient was newly diagnosed with DM during the current study.

The mean duration of dapsone intake was 30 weeks. When stratified into different weeks, 8 patients were taking it for less than 12 weeks; 6 were taking it for 12 - 24 weeks, 9 for 25 - 48 weeks, and 7 for more than 48 weeks. Among those on dapsone therapy for less than 12 weeks, three out of nine (30%) had high methemoglobin levels. However, only one patient had high methemoglobin levels (1 out of 21) among those on dapsone for more than 12 weeks. This implies that methemoglobin probably occurs during early therapy and resolves as the patients remain on dapsone therapy for a longer duration. However, MCV and LDH levels did not show any difference between the dapsone treatment-stratified groups and were equally distributed between the groups, suggesting that hemolysis is dependent on the duration of therapy. A stepwise regression analysis was performed to assess the effects of various factors associated with discordant HbA1c in those receiving dapsone therapy. Of all the factors studied (age, BMI, gender, duration of dapsone therapy, reticulocyte, MCV, LDH, and methemoglobin), the standardized coefficient beta for age was 0.5, demonstrating that younger age was associated with a greater reduction in HbA1c levels (p-0.01). All other parameters did not influence the change in HbA1c levels.

## 5. Discussion

Glycated hemoglobin (HbA1c) is an important tool, both as a diagnostic marker for DM and as a marker for long-term glycemic control. There are various drugs as well as conditions that can alter HbA1c levels, thereby resulting in misleading values that may adversely affect diabetes management. Important conditions known to influence HbA1c include hemolysis, methemoglobinemia, and hemoglobinopathies. Several drugs can also alter HbA1c levels, one such drug being dapsone, which is commonly used in the treatment of Hansen’s disease (23). Although the association between dapsone use and inappropriately low HbA1c levels has been described previously, the existing literature largely consists of isolated case reports and small case series. In our previous study evaluating discordant HbA1c in patients receiving dapsone therapy, discordance was defined by a mean difference of more than 28.7 mg/dL (corresponding to a 1% change in HbA1c) between estimated average glucose (eAG) derived from measured HbA1c and mean blood glucose values obtained through self-monitoring of blood glucose (SMBG). Concordance was defined as a mean difference of less than 28.7 mg/dL. However, in the current study, discordant and concordant HbA1c values were defined using the glycosyl gap, calculated as the difference between measured HbA1c and expected HbA1c derived from fructosamine.

**Figure 1.**
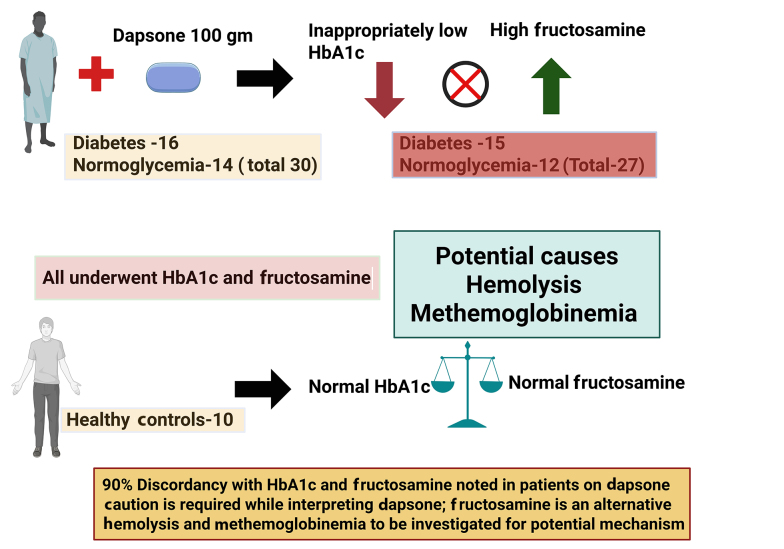
Discordantly low HbA1c in dapsone-treated patients despite elevated fructosamine: Role of hemolysis and methemoglobinemia

The prevalence of discordant HbA1c in the present study was 90%, which is higher than that observed in our previous study (71%) (Figure 1). 

This difference may be related to differences in study design, definitions of discordance, and glycemic markers used for comparison. The available literature on this topic remains limited. Shah et al. documented a reduction in HbA1c levels following initiation of dapsone therapy, with normalization after drug withdrawal (24). Similarly, Unnikrishnan et al. reported low HbA1c levels in two patients with type 2 DM receiving dapsone (16). In our study, 27 out of 30 patients (90%) demonstrated discordantly low HbA1c values, which was statistically significant (P = 0.01). Among patients with DM, 15 of 16 (94%) had discordant HbA1c values, and among normoglycemic individuals, 12 of 14 also had discordantly low HbA1c values when compared with fructosamine and glycosyl gap assessments (Table 4) (12). The study population represents patients receiving dapsone therapy for Hansen’s disease at a single tertiary care center, identified through consecutive inclusion during retrospective chart review. While this cohort is representative of patients treated with dapsone in similar tertiary care settings, it may not fully represent all patients with DM receiving dapsone in other clinical or geographic contexts. Therefore, caution is warranted when generalizing these findings beyond comparable populations.

A limitation of this study is the choice of control groups. Although diabetic patients not receiving dapsone would represent an ideal comparator for certain analyses, healthy controls were intentionally selected to establish baseline glycosyl gap and hemolysis-related parameters in the absence of both dapsone exposure and diabetes-related confounders. Diabetes mellitus itself may influence erythrocyte turnover, oxidative stress, and hemoglobin glycation, which could interfere with the interpretation of mechanistic markers such as hemolysis and methemoglobinemia. The absence of a diabetic non-dapsone control group limits direct causal inference, and future prospective studies incorporating appropriately matched diabetic control groups are required to further delineate the independent effects of dapsone on HbA1c. Some investigators have reported anemia with elevated reticulocyte counts and LDH levels in patients receiving dapsone, suggesting hemolysis as a possible mechanism. Additionally, elevated methemoglobin levels have been reported, indicating another potential contributor to spuriously low HbA1c values (16). In our study, MCV and LDH levels were higher in patients with discordant HbA1c, suggesting a trend toward hemolysis. However, these differences did not reach statistical significance. Thus, while hemolysis and methemoglobinemia remain biologically plausible mechanisms, our findings should be interpreted as hypothesis-generating rather than confirmatory.

It should be noted that subgroup analyses, particularly comparisons involving the concordant HbA1c group, were limited by very small sample sizes. Consequently, these analyses were exploratory in nature and intended to describe observed trends rather than to support definitive statistical or mechanistic conclusions. Elevated methemoglobin levels have been reported in patients with DM receiving dapsone therapy. In our cohort, methemoglobin levels were marginally higher in patients with discordant HbA1c compared to those with concordant HbA1c; however, this difference was not statistically significant. Thus, while hemolysis and methemoglobinemia remain biologically plausible mechanisms, our findings should be considered hypothesis-generating rather than confirmatory. As both hemolysis and methemoglobinemia can affect HbA1c estimation, alternative methods may be required to assess long-term glycemic control in patients receiving dapsone therapy. Fructosamine represents a useful alternative marker, although it reflects glycemic control over a shorter duration of approximately three weeks (25). In our study, 15 of 16 patients with DM had low HbA1c values despite elevated fructosamine levels, supporting the clinical relevance of alternative glycemic markers in this setting.

The principal limitation of our study is the relatively small sample size, which was calculated to estimate prevalence rather than to provide sufficient power for robust subgroup or mechanistic analyses. As a result, conclusions regarding the underlying mechanisms of discordant HbA1c should be interpreted with caution. Additional limitations include the retrospective design and the single-center nature of the study, which may limit generalizability. Future large-scale, prospective studies with appropriately matched diabetic control groups and standardized assessment of dapsone dose and duration are needed to confirm these findings and further elucidate the underlying mechanisms.

### 5.1. Conclusions

In conclusion, this study demonstrates a high prevalence of discordantly low HbA1c values among patients receiving dapsone therapy, underscoring an important clinical pitfall in the interpretation of HbA1c in this setting. Dapsone may interfere with HbA1c estimation even in the absence of overt hematological abnormalities or clinical symptoms, potentially due to subclinical effects on erythrocyte turnover and oxidative hemoglobin changes.

Although trends toward hemolysis and elevated methemoglobin levels were observed, these findings did not reach statistical significance and therefore cannot be considered definitive mechanisms based on the current data. Accordingly, hemolysis and methemoglobinemia should be regarded as biologically plausible but unproven contributors, warranting further investigation. Physicians should be aware that spuriously low HbA1c values may be misleading in patients treated with dapsone, particularly when capillary or laboratory glucose measurements suggest poor glycemic control. A careful medication history remains crucial in avoiding misinterpretation of HbA1c values. Alternative glycemic markers such as fructosamine or 1,5-anhydroglucitol may be useful adjuncts for monitoring glycemic control in patients receiving dapsone. Given the small sample size and exploratory nature of mechanistic analyses, larger prospective studies are required to confirm these observations and to clarify the underlying biological mechanisms.

## Data Availability

The dataset presented in the study is available on request from the corresponding author during submission or after publication. The data are not publicly available due to institutional Policy.
